# Ratios of Neutrophils and Platelets to Lymphocytes as Predictors of Postoperative Intensive Care Unit Admission and Length of Stay in Bariatric Surgery Patients: A Retrospective Study

**DOI:** 10.3390/medicina60050753

**Published:** 2024-04-30

**Authors:** Mohmad H. Alsabani, Faraj K. Alenezi, Badi A. Alotaibi, Ahmed A. Alotaibi, Lafi H. Olayan, Saleh F. Aljurais, Najd Alarfaj, Deem Alkhurbush, Ghaida Almuhaisen, Lena Alkhmies, Mohammed K. Al Harbi

**Affiliations:** 1 Anesthesia Technology Department, College of Applied Medical Sciences, King Saud bin Abdulaziz University for Health Sciences, Riyadh 11481, Saudi Arabia; alenezifa@ksau-hs.edu.sa (F.K.A.); .; olayanl@ksau-hs.edu.sa (L.H.O.); juraiss@ksau-hs.edu.sa (S.F.A.); alarfaj162@ksau-hs.edu.sa (N.A.); alkhurbush014@ksau-hs.edu.sa (D.A.); almuhaisen051@ksau-hs.edu.sa (G.A.); alkhmies154@ksau-hs.edu.sa (L.A.); 2 King Abdullah International Medical Research Centre, Riyadh 11481, Saudi Arabia; otaibibad@ksau-hs.edu.sa (B.A.A.); harbimk@ngha.med.sa (M.K.A.H.); 3 Clinical Laboratory Sciences Department, College of Applied Medical Sciences, King Saud bin Abdulaziz University for Health Sciences, Riyadh 11481, Saudi Arabia; 4 Department of Anesthesia, Ministry of National Guard Health Affairs, Riyadh 11426, Saudi Arabia; 5 College of Medicine, King Saud bin Abdulaziz University for Health Sciences, Riyadh 11481, Saudi Arabia

**Keywords:** bariatric surgery, NLR, PLR, immune-inflammation, length of stay, ICU admission

## Abstract

*Background and Objectives*: This study aimed to investigate the role of the pre- and postoperative neutrophil-to-lymphocyte ratio (NLR) and platelet-to-lymphocyte ratio (PLR) in predicting intensive care unit (ICU) admission and postoperative length of stay (LOS) in bariatric surgery. *Materials and Methods*: We retrospectively analysed 96 patients who underwent bariatric surgery at our institution. The NLR and PLR were calculated in the pre- and postoperative stages. Changes in pre- and postoperative hematological ratios were compared using the Wilcoxon signed-rank test. The optimal cutoff values and area under the curve (AUC) for each ratio were calculated using receiver operating characteristic (ROC) analysis. Multivariate linear regression analysis was used to assess the relationship between each ratio and the postoperative LOS after adjusting for age, sex, and American Society of Anesthesiologists (ASA) score. *Results*: The median age of our patients was 35.50 years, and 54.2% were male. The preoperative NLR showed a significant increase from 1.44 to 6.38 postoperatively (*p* < 0.001). The PLR increased from 107.08 preoperatively to 183.58 postoperatively, *p* < 0.001). ROC analysis showed that the postoperative NLR was a moderate to high predictor of ICU admission (AUC = 0.700, optimal cutoff point = 5.987). The postoperative PLR had less predictive power for ICU admission (AUC = 0.641, optimal cutoff point = 170.950). Ratios that had a statistically significant relationship with the postoperative LOS were the preoperative NLR (standardized β [95% CI]: 0.296 [0.115–0.598]), postoperative NLR (0.311 [0.034–0.161]), and postoperative PLR (0.236 [0.000–0.005]). *Conclusions*: The NLR and PLR demonstrated an independent relationship with the postoperative LOS after bariatric surgery and the predictive ability of ICU admission. Both ratios might be useful as simple markers to predict patient outcome after surgery.

## 1. Introduction

Overweight and obesity are significant global health challenges, and their prevalence has escalated markedly since 1975. The World Health Organization reports that 39% of adults are overweight (BMI > 25 kg/m^2^) and 13% are obese (BMI > 30 kg/m^2^), affecting over 1.9 billion individuals globally. These conditions are independent risk factors for various health issues, including cardiovascular diseases, musculoskeletal disorders, such as osteoarthritis, metabolic diseases, such as diabetes, and certain types of cancer [1].

Management strategies for obesity are multifaceted and vary based on the severity of obesity, associated chronic illnesses, and genetic factors. Lifestyle modification is the primary approach; however, for individuals with morbid obesity (BMI > 40 kg/m^2^) or those with a BMI > 35 kg/m^2^ with associated comorbidities such as diabetes and sleep apnea, bariatric surgery is often the recommended course [2]. Bariatric procedures, including Roux-en-Y gastric bypass, adjustable gastric banding, and sleeve gastrectomy, are increasingly being utilized, with over 340,000 surgeries performed globally [3]. Therefore, identifying patients at a higher risk of early and late postoperative complications associated with these surgeries is crucial [4].

Obesity significantly contributes to inflammation, which is closely associated with changes in insulin resistance, atherosclerosis, hypertension, and certain cancers [5,6]. Overweight and obese individuals exhibit variations in serum levels of inflammatory cytokines including tumor necrosis factor-alpha (TNF-α), C-reactive protein (CRP), interleukins-6 (IL-6), and interleukins-18 (IL-18) [7,8,9,10]. It has been observed that body fat measurements show a stronger correlation with these inflammatory markers than BMI [8,10,11]. Although CRP and IL-6 are valuable indicators of systemic inflammation in bariatric surgery, they are not routinely investigated in the perioperative period among bariatric patients [12].

Inflammation following major surgeries is crucial and is balanced by pro- and anti-inflammatory responses within the innate and adaptive immune systems, which promote repair and healing [13,14]. However, a dysregulated inflammatory response can elevate the risk of organ dysfunction and postoperative complications, and lead to poorer recovery outcomes, including persistent disability or death [15,16,17,18]. A recent study by Bain et al. (2023) showed that increased levels of postoperative systemic inflammation are significantly associated with a higher risk of serious complications, including unplanned intensive care unit (ICU) admissions, prolonged ICU and hospital stays, and 90-day mortality after major abdominal surgery [19]. This highlights the importance of inflammatory markers in improving patient-centered outcomes.

In the context of bariatric surgery, hematological inflammatory markers such as the neutrophil-to-lymphocyte ratio (NLR) have gained attention for their predictive and prognostic values [20,21]. These markers are easily obtainable from routine complete blood count (CBC) analyses and provide cost-effective and readily available data for perioperative risk assessment. Recent studies, including those by Aykota et al. (2021) and Ertugrul and Kuzu (2019), have demonstrated significant decreases in the NLR value 6-months and 1-year following bariatric procedures such as laparoscopic sleeve gastrectomy, correlating with reductions in BMI. Aykota et al. also observed significant changes in the platelet-to-lymphocyte ratio (PLR) value 1-year after surgery, adding further insight into the role of these markers in tracking long-term surgical outcomes and patient recovery [22,23].

The roles of the NLR and PLR are well established in non-bariatric surgeries such as cardiac surgery, where they have been found to predict 90-day mortality and length of hospital stay [24]. Notably, Tzikos et al. (2023) revealed that higher postoperative NLR values and longer ICU lengths of stay were independent risk factors for 90-day mortality in cardiac surgery patients [24]. Moreover, the preoperative NLR has been reported to correlate with the Clavien–Dindo classification system of postoperative complications in acute calculous cholecystitis patients requiring surgical treatment [25]. This finding highlights the importance of these markers in critical care settings. However, the specific roles of the NLR and PLR as predictive tools in the perioperative period of bariatric surgery remain underexplored, particularly in predicting postoperative complications, such as ICU admission. This gap highlights the need for further investigation of the utility of these inflammatory markers in a bariatric surgery setting.

Therefore, our study aimed to investigate the predictive value of the NLR and PLR in bariatric surgery. We focused on the postoperative outcomes, with the primary endpoint being ICU admission and the secondary endpoint being the length of hospital stay. By providing a clearer understanding of these markers in the context of bariatric surgery, our research seeks to contribute to the optimization of patient care and management in this increasingly prevalent surgical field.

## 2. Materials and Methods

This retrospective cohort study was conducted at King Abdulaziz Medical City (KAMC) in Riyadh, Saudi Arabia. The study was approved by the Institutional Review Board (IRB) of the King Abdullah International Medical Research Centre (KAIMRC) (IRB/1569/22). The need for informed consent was waived by the IRB at KAIMRC due to the retrospective nature of the study. The study was conducted in accordance with the Declaration of Helsinki and local and institutional guidelines.

Patients included in this study (1) were older than 18 years, (2) had undergone bariatric surgery, and (3) had complete medical records. Patients were excluded from this study if they (1) were younger than 18 years, (2) had incomplete medical records, (3) had undergone non-bariatric surgery, (4) had previous malignancies, and (5) had coagulopathies. The primary outcome was postoperative ICU admission and the secondary outcome was hospital length of stay (LOS), which was defined as the number of days from the patient’s postoperative admission until discharge.

The following laboratory and clinical information was collected: sociodemographic data including sex, age, and BMI, along with clinical data including the American Society of Anesthesiologists (ASA) score, Obstructive Sleep Apnea (OSA), and surgery time (without and with anesthesia time). Pre- and postoperative laboratory parameters were obtained, including lymphocyte and neutrophil counts, platelet counts, white blood cell count, and hemoglobin levels. Postoperative CBC data were eligible for analysis if performed within 7 days after surgery since there is no standardized protocol on the timing of postoperative blood collection in our hospital. The following hematological parameters were calculated for all patients, including the NLR, defined as the ratio between absolute neutrophil count and absolute lymphocyte count, and the PLR, defined as the ratio between absolute platelet count and absolute lymphocyte count. Postoperative outcomes, including ICU admission, reason for ICU admission, and the postoperative LOS, were obtained. All data were extracted from the electronic medical records database of KAMC. The reasons for ICU admission were stratified based on the cause of ICU admission.

The data in this study were analyzed using SPSS (version 28.0; IBM, Armonk, NY, USA). Categorical parameters are presented as frequencies and percentages. Continuous variables were checked for normality using the Shapiro–Wilk test. All clinical data were non-normally distributed and are presented as medians (interquartile range). Laboratory parameters before and after surgery were compared using the Wilcoxon signed-rank test. Receiver operating characteristic (ROC) curve analysis was performed to identify the ability of the pre- and postoperative NLR and PLR to predict ICU admission. The area under the curve (AUC), cutoff value, sensitivity, and specificity were calculated. Univariate and multivariate linear regression analyses were performed to examine the influence of the NLR and PLR on the postoperative LOS. The multivariate analysis was adjusted for age, sex, and ASA classification score. Variables in the NLR and PLR formulas were not included in multivariate analysis. The level of significance was set at *p* < 0.05 (two-sided).

## 3. Results

As shown in Table 1, the participants included 52 men (54.2%) and 44 women (45.83%), with a median age of 35.50 years. All patients underwent laparoscopic sleeve gastrectomy. The majority of our patients were categorized by the ASA classification system as ASA III (49%), followed by ASA II (41.7%). Thirty-three patients (34.4%) had a history of endocrine comorbidities and 30 (31.3%) had a history of cardiovascular comorbidities. In addition, only seven patients had a history of OSA. Of the 96 patients, only 12 (12.5%) required ICU admission, and cardiovascular complications (41.6%) were the dominant cause of ICU admission. The median length of hospital stay in the entire cohort was 4.00 days.

Next, we analyzed the differences between the preoperative and postoperative laboratory parameters and hematological ratios (Table 2). The Wilcoxon signed-rank test indicated a significant difference between pre- and postoperative neutrophil counts (3.87 × 10^9^/L vs. 9.54 × 10^9^/L, *p* < 0.001). A marked increase was also observed in WBC count from 7.40 × 10^9^/L in the preoperative stay to 11.75 × 10^9^/L in the postoperative stay (*p* < 0.001). On the other hand, we observed a significant decrease in lymphocyte count from 2.82 × 10^9^/L preoperative to 1.60 × 10^9^/L postoperatively (*p* < 0.001). A slight but significant decrease in hemoglobin levels was also noted between the pre- and postoperative periods (133.50 vs. 130.00, *p* < 0.001). Importantly, both the NLR and PLR markedly increased from 1.44 and 107.08 in the preoperative period to 6.38 and 183.58, respectively (all *p* < 0.001).

As shown in Table 3, ROC analysis demonstrated that the postoperative NLR had the strongest predictive power with 91.7% sensitivity, 51.2% specificity, and an optimal cutoff point of 5.987 at AUC = 0.700, 95% CI = 0.583–0.818. (The higher the AUC-ROC value, the stronger the biomarker’s predictivity for ICU admission.) The preoperative NLR had a modest predictive ability (AUC = 0.642, 95% CI = 0.465–0.819), with 75% sensitivity, 52.4% specificity, and an optimal cutoff point of 1.358. The PLR was generally less predictive of ICU admissions based on ROC analysis than the NLR. The postoperative PLR, with 91.7% sensitivity, 52.4% specificity, and an optimal cutoff point of 170.950, had an AUC of 0.641 (95% CI = 0.506–0.776. The lowest predictive ability corresponded to the preoperative PLR at AUC = 0.629, 95% CI = 0.476–0.782, with 83.3% sensitivity, 60.7% specificity, and an optimal cutoff point of 93.285. (Table 3).

We assessed the association between various factors and the length of hospital stay using univariate linear regression analysis. (Table 4). The LOS was associated with the following: the preoperative NLR (β = 0.292, 95% CI 0.116–0.588, *p* = 0.004), postoperative lymphocyte count (β = −0.366, 95% CI −0.959–−0.302, *p* < 0.001), postoperative NLR (β = 0.293, 95% CI 0.030–0.153, *p* = 0.004), and postoperative PLR (*p* = 0.027). We then performed multivariable linear regression analysis to identify the independent impact of the hematological ratios on the postoperative LOS (Table 5). We found that the postoperative LOS was independently associated with the preoperative NLR (β = 0.296, 95% CI 0.115–0.598, *p* = 0.004), postoperative NLR (β = 0.311, 95% CI 0.034–0.161, *p* = 0.003), and postoperative PLR (β = 0.236, 95% CI 0.000–0.005, *p* = 0.024).

## 4. Discussion

As operating room practitioners, including surgeons and anesthetists, are required to assist in identifying potential adverse outcomes in the early perioperative stages, inflammatory indicators demonstrated their effectiveness during this time. Simple and inexpensive parameters, such as the NLR and PLR, can aid surgeons in early identification, lowering death rates, and improving discharge times. Elevation of these hematological ratios in surgical patients is clinically important as it indicates an ongoing inflammatory process and arguably has prognostic implications. In this study, the NLR and PLR exhibited significant differences across two time points: the immediate preoperative and postoperative periods. Our findings highlight that the NLR and PLR are valuable parameters for predicting ICU admission. In univariate analysis, the preoperative NLR and postoperative NLR and PLR were associated with the postoperative LOS. This significance was maintained in multivariate analysis after adjusting for age, sex, and ASA classification score.

Because of their ability to identify cases of high physiological stress, the NLR and PLR have been explored as markers of severity and prognosis. They have been widely used in a variety of diseases and disorders, including acute pancreatitis [26], acute coronary syndrome [27], acute pulmonary embolism [28], and rheumatic diseases [29]. Importantly, the performance of these hematological indices was similar to that of CRP [30,31]. Previous research has shown that the NLR is a useful prognostic marker for individuals with sepsis [32,33] and critically ill populations in general [34]. Due to its remarkable diagnostic accuracy in sepsis, pneumonia, and bacteremia, the NLR has drawn a lot of attention [35]. The NLR is at least a moderate predictor of bacteremia, with AUROCs ranging from 0.7 to 0.77 [36]. The NLR has a strong association and equivalent effectiveness in identifying bacterial sepsis in emergency care settings when compared to other biomarkers, such as CRP and procalcitonin. Regardless of the microbiological cause, the NLR may represent a patient’s physiological stress when they are critically unwell [37]. Our results are supported by those of previous studies. Although ICU admission after bariatric surgery was uncommon in the current study, our findings demonstrated that the NLR and PLR have potential predictive abilities for ICU admission in bariatric surgery patients. In line with our findings, Da Silva et al. found that the postoperative NLR was significantly associated with major complications such as unplanned intubation [21]. However, the value of the PLR in predicting major complications was not explored in the same study. The fundamental functions of platelets in maintaining hemostasis are essential for adaptive immunity and the initiation of an inflammatory response [38]. For example, a significant relationship was found between hospital mortality and platelet count in patients with community-acquired pneumonia [39]. The PLR has been demonstrated to have good predictive value in individuals with cancers [40,41], acute myocardial infarction [42], or stable coronary artery disease [43]. Studies demonstrating a relationship between the PLR and ICU duration of stay [44], and even hospital mortality [45,46], demonstrate how its application in prognostication has expanded to the critically ill population.

Obesity-induced inflammation is common and has long been recognized to be associated with adverse outcomes such as the development of type 2 diabetes, coronary artery calcification, and stroke [47,48,49]. Inflammatory markers measured in obesity-related studies often include IL-6, TNF-α, and CRP [12]. Although these biomarkers are valuable indicators of systemic inflammation, they are not routinely used in the clinical setting. Currently, research efforts are shifting towards the development and validation of simple and inexpensive biomarkers. Immune-inflammatory markers, such as the NLR and PLR, are used to forecast the immune-inflammatory process, which was found to be a useful criterion for determining which patients are at risk for morbidities and postoperative adverse outcomes [21]. It is important to note that while clinical findings are the best way to identify potential adverse events, these predictive biomarkers may be crucial in identifying complications following surgery, especially when symptoms are not present. This study confirms and extends previous studies showing that the evaluation of immune-inflammatory markers, such as the NLR and PLR, is crucial. In support of this, we showed a marked increase in both the NLR and PLR measured at two time points, before and after surgery. An increased NLR and PLR reflect the degree of activated immune-inflammatory response and, thus, vulnerability to adverse outcomes after surgery. Since obesity is a well-known pro-inflammatory condition, similar to cancer patients [50,51], our study demonstrated the value of measuring the NLR and PLR at two different time points and showed the potential to predict ICU admission and longer hospitalization after surgery.

Although blood measurements are frequently utilized as prognostic values for many diseases and cancers, their effect on the development of complications in ICU patients remains uncertain. The results revealed significant changes in neutrophils, lymphocytes, WBC, hemoglobin, the NLR, and the PLR in the preoperative and postoperative periods, demonstrating how the inflammatory response plays a role in complications. In fact, preoperative monitoring of these simple markers can help clinicians to identify high-risk patients and develop optimization strategies. In addition, longitudinal analysis of these markers during the perioperative stage may allow clinicians to personalize patient care and early detection of postoperative complications. Although this study contains useful information, it has numerous limitations, most notably, its retrospective approach. Furthermore, there was no definite blood collection time for postoperative hematological examinations. Other limitations include the relatively small number of cases, and more prospective studies with larger numbers of patients are needed to expand our understanding of this area.

## 5. Conclusions

Currently, the risk assessment of patients undergoing bariatric surgery is based on clinical evaluations. As the NLR and PLR are simple and inexpensive parameters that can be easily acquired from complete blood count analysis, they have the potential to be incorporated into the daily clinical practice of bariatric surgery patients. Based on our findings, it is evident that the NLR and PLR can serve as reliable parameters to predict ICU admission and are independently associated with postoperative hospital LOS. Our findings highlight the potential clinical significance of these ratios in identifying high-risk patients and in optimizing postoperative care. However, further large-scale prospective studies are necessary to validate our findings and assess their usefulness in comparison to other inflammatory parameters such as CRP.

## Figures and Tables

**Table 1:** Baseline and postoperative outcomes of the entire cohort.

Variable	All (n = 96)
Age, years, median (IQR)	35.50 (16.50)
Gender, Male, *n* (%)	52 (54.2)
BMI, Kg/m^2^, median (IQR)	44.10 (6.92)
OSA, *n* (%)	7 (7.30)
ASA, *n* (%)	
ASA I	7 (7.3)
ASA II	40 (41.7)
ASA III	47 (49.0)
ASA IV	2 (2.1)
Comorbidity, *n* (%)	
Respiratory	0 (0)
Endocrine	33 (34.4)
Cardiovascular	30 (31.3)
Duration of surgery, minutes, median (IQR)	
Without anesthesia	77.50 (45.50)
Including anesthesia	105.00 (52.75)
Admission to ICU	12 (12.5)
Reason for ICU admission, *n* (%)	
Cardiovascular	5 (41.6)
Respiratory	2 (16.7)
Hematological	1 (8.3)
Others	4 (33.3)
Postoperative LOS, days, median (IQR)	4.00 (1.00)

BMI, body mass index; OSA, obstructive sleep apnea; ASA, American Society of Anesthesiologists score; LOS, length of stay. Continuous data are presented as the median (interquartile range), and categorical data are presented as number of cases (%).

**Table 2:** Changes in laboratory values and hematological ratios before and after surgery.

Variable	Preoperative Value	Postoperative Value	*z*	*p*-Value
Neutrophils (×10^9^/L)	3.87	9.54	−8.370	<0.001 *
Platelets (×10^9^/L)	288.00	289.50	−0.202	0.840 *
Lymphocytes (×10^9^/L)	2.82	1.60	−8.233	<0.001 *
WBC (×10^9^/L)	7.40	11.75	−7.845	<0.001 *
Hemoglobin	133.50	130.00	−5.281	<0.001 *
NLR	1.44	6.38	−8.409	<0.001 *
PLR	107.08	183.58	−7.886	<0.001 *

BMI, body mass index; OSA, obstructive sleep apnea; ASA, American Society of Anesthesiologists score; LOS, length of stay. * Statistically significant findings from Wilcoxon signed-rank test.

**Table 3:** Receiver operating characteristic analysis of hematological ratios for intensive care unit admission.

Variable	AUC	95% CI	Optimal Cutoff Point	Sensitivity	Specificity
Preoperative NLR	0.642	0.465–0.819	1.358	0.750	0.524
Postoperative NLR	0.700	0.583–0.818	5.987	0.917	0.512
Preoperative PLR	0.629	0.476–0.782	93.285	0.833	0.607
Postoperative PLR	0.641	0.506–0.776	170.950	0.917	0.524

NLR, neutrophil-to-lymphocyte ratio; PLR, platelet-to-lymphocyte ratio.

**Table 4:** Univariable linear regression analysis of the factors associated with postoperative length of hospital stay.

Variable	Standardized β [95% CI]	*p*-Value
Age	0.040 [−0.017–0.025]	0.696
Gender	−0.016 [−0.543–0.467]	0.880
BMI	0.038 [−0.037–0.054]	0.713
ASA	0.192 [−0.307–0.455]	0.702
Preoperative laboratory values		
Platelets	−0.110 [−0.005–0.002]	0.285
Neutrophils	0.049 [−0.125–0.203]	0.639
Lymphocytes	−0.189 [−0.714–0.021]	0.065
NLR	0.292 [0.116–0.588]	0.004 *
PLR	0.086 [−0.004–0.010]	0.405
WBC	−0.060 [−0.188–0.102]	0.559
Hemoglobin	−0.009 [−0.016–0.015]	0.933
Postoperative laboratory values		
Platelets	−0.081 [−0.004–0.002]	0.434
Neutrophils	0.137 [−0.024–0.127]	0.182
Lymphocytes	−0.366 [−0.959–−0.302]	<0.001 *
NLR	0.293 [0.030–0.153]	0.004 *
PLR	0.225 [0.000–0.005]	0.027 *
WBC	0.058 [−0.051–0.091]	0.576
Hemoglobin	−0.055 [−0.020–0.011]	0.597
Duration of surgery (without anesthesia)	0.064 [−0.005–0.010]	0.537
Duration of surgery (with anesthesia)	0.131 [−0.002–0.011]	0.203

CI, confidence interval; BMI, body mass index; ASA, American Society of Anesthesiologists score; NLR, neutrophil-to-lymphocyte ratio; PLR, platelet-to-lymphocyte ratio; WBC, white blood cell. * Statistically significant results.

**Table 5:** Multivariable linear regression analysis of the independent relationship between hematological ratios and postoperative length of hospital stay.

Variable	Standardized β [95% CI]	*p*-Value
Preoperative NLR	0.296 [0.115–0.598]	0.004 *
Postoperative NLR	0.311 [0.034–0.161]	0.003 *
Preoperative PLR	0.095 [−0.004–0.011]	0.368
Postoperative PLR	0.236 [0.000–0.005]	0.024 *

NLR, neutrophil-to-lymphocyte ratio; PLR, platelet-to-lymphocyte ratio. * Statistically significant results.

## Data Availability

The data that support the findings of this study are available from KAIMRC, but restrictions apply to the availability of these data, which were used under license for the current study, and so are not publicly available.

## References

[1] World Health Organization (2017). Obesity and Overweight.

[2] Busetto L., Dixon J., De Luca M., Shikora S., Pories W., Angrisani L. (2014). Bariatric surgery in class I obesity: A Position Statement from the International Federation for the Surgery of Obesity and Metabolic Disorders (IFSO). Obes. Surg..

[3] Buchwald H., Oien D.M. (2013). Metabolic/bariatric surgery worldwide 2011. Obes. Surg..

[4] Lim R., Beekley A., Johnson D.C., Davis K.A. (2018). Early and late complications of bariatric operation. Trauma Surg. Acute Care Open.

[5] Bastard J.-P., Maachi M., Lagathu C., Kim M.J., Caron M., Vidal H., Capeau J., Feve B. (2006). Recent advances in the relationship between obesity, inflammation, and insulin resistance. Eur. Cytokine Netw..

[6] Hotamisligil G.S. (2006). Inflammation and metabolic disorders. Nature.

[7] Bulló M., García-Lorda P., Megias I., Salas-Salvadó J. (2003). Systemic inflammation, adipose tissue tumor necrosis factor, and leptin expression. Obes. Res..

[8] Festa A., D’Agostino Jr R., Williams K., Karter A., Mayer-Davis E., Tracy R., Haffner S. (2001). The relation of body fat mass and distribution to markers of chronic inflammation. Int. J. Obes..

[9] Mabrouk R., Ghareeb H., Shehab A., Omar K., El-Kabarity R.H., Soliman D.A., Mohamed N.A. (2013). Serum visfatin, resistin and IL-18 in A group of Egyptian obese diabetic and non diabetic individuals. Egypt. J. Immunol..

[10] Park H.S., Park J.Y., Yu R. (2005). Relationship of obesity and visceral adiposity with serum concentrations of CRP, TNF-α and IL-6. Diabetes Res. Clin. Pract..

[11] Baetge C., Earnest C.P., Lockard B., Coletta A.M., Galvan E., Rasmussen C., Levers K., Simbo S.Y., Jung Y.P., Koozehchian M. (2017). Efficacy of a randomized trial examining commercial weight loss programs and exercise on metabolic syndrome in overweight and obese women. Appl. Physiol. Nutr. Metab..

[12] Rodríguez-Hernández H., Simental-Mendía L.E., Rodríguez-Ramírez G., Reyes-Romero M.A. (2013). Obesity and inflammation: Epidemiology, risk factors, and markers of inflammation. Int. J. Endocrinol..

[13] Alazawi W., Pirmadjid N., Lahiri R., Bhattacharya S. (2016). Inflammatory and immune responses to surgery and their clinical impact. Ann. Surg..

[14] Watanabe S., Alexander M., Misharin A.V., Budinger G.S. (2019). The role of macrophages in the resolution of inflammation. J. Clin. Investig..

[15] Dobson G.P. (2020). Trauma of major surgery: A global problem that is not going away. Int. J. Surg..

[16] Ferraris V.A., Ballert E.Q., Mahan A. (2013). The relationship between intraoperative blood transfusion and postoperative systemic inflammatory response syndrome. Am. J. Surg..

[17] Lahiri R., Derwa Y., Bashir Z., Giles E., Torrance H.D., Owen H.C., O’Dwyer M.J., O’Brien A., Stagg A.J., Bhattacharya S. (2016). Systemic inflammatory response syndrome after major abdominal surgery predicted by early upregulation of TLR4 and TLR5. Ann. Surg..

[18] Story D.A., Leslie K., Myles P.S., Fink M., Poustie S.J., Forbes A., Yap S., Beavis V., Kerridge R., on behalf of the REASON Investigators, Australian and New Zealand College of Anaesthetists Trials Group (2010). Complications and mortality in older surgical patients in Australia and New Zealand (the REASON study): A multicentre, prospective, observational study. Anaesthesia.

[19] Bain C.R., Myles P.S., Martin C., Wallace S., Shulman M.A., Corcoran T., Bellomo R., Peyton P., Story D.A., Leslie K. (2023). Postoperative systemic inflammation after major abdominal surgery: Patient-centred outcomes. Anaesthesia.

[20] Zubiaga L., Ruiz-Tovar J. (2020). Correlation of preoperative neutrophil-to-lymphocyte ratio and platelet-to-lymphocyte ratio with metabolic parameters in patients undergoing sleeve gastrectomy. Surg. Obes. Relat. Dis..

[21] Da Silva M., Cleghorn M.C., Elnahas A., Jackson T.D., Okrainec A., Quereshy F.A. (2017). Postoperative day one neutrophil-to-lymphocyte ratio as a predictor of 30-day outcomes in bariatric surgery patients. Surg. Endosc..

[22] Aykota M.R., Yilmaz S., Atabey M., Ozgen U., Simsek S. (2021). Effect of Sleeve Gastrectomy on the Neutrophil-to-Lymphocyte Ratio, the Platelet-to-Lymphocyte Ratio, Platelet Counts, and Mean Platelet Volumes. Indian J. Surg..

[23] Ertugrul I., Kuzu F. (2019). The impact of bariatric surgery on hematological inflammatory parameters. Ann. Med Res..

[24] Tzikos G., Alexiou I., Tsagkaropoulos S., Menni A.E., Chatziantoniou G., Doutsini S., Papavramidis T., Grosomanidis V., Stavrou G., Kotzampassi K. (2023). Neutrophil-to-Lymphocyte Ratio and Platelet-to-Lymphocyte Ratio as Predictive Factors for Mortality and Length of Hospital Stay after Cardiac Surgery. J. Pers. Med..

[25] Serban D., Stoica P.L., Dascalu A.M., Bratu D.G., Cristea B.M., Alius C., Motofei I., Tudor C., Tribus L.C., Serboiu C. (2023). The Significance of Preoperative Neutrophil-to-Lymphocyte Ratio (NLR), Platelet-to-Lymphocyte Ratio (PLR), and Systemic Inflammatory Index (SII) in Predicting Severity and Adverse Outcomes in Acute Calculous Cholecystitis. J. Clin. Med..

[26] Kaplan M., Ates I., Oztas E., Yuksel M., Akpinar M.Y., Coskun O., Kayacetin E. (2018). A new marker to determine prognosis of acute pancreatitis: PLR and NLR combination. J. Med. Biochem..

[27] Li Q., Ma X., Shao Q., Yang Z., Wang Y., Gao F., Zhou Y., Yang L., Wang Z. (2022). Prognostic impact of multiple lymphocyte-based inflammatory indices in acute coronary syndrome patients. Front. Cardiovasc. Med..

[28] Ma Y., Mao Y., He X., Sun Y., Huang S., Qiu J. (2016). The values of neutrophil to lymphocyte ratio and platelet to lymphocyte ratio in predicting 30 day mortality in patients with acute pulmonary embolism. BMC Cardiovasc. Disord..

[29] Gasparyan A.Y., Ayvazyan L., Mukanova U., Yessirkepov M., Kitas G.D. (2019). The platelet-to-lymphocyte ratio as an inflammatory marker in rheumatic diseases. Ann. Lab. Med..

[30] de Jager C.P., Wever P.C., Gemen E.F., Kusters R., van Gageldonk-Lafeber A.B., van der Poll T., Laheij R.J. (2012). The neutrophil-lymphocyte count ratio in patients with community-acquired pneumonia. PLoS ONE.

[31] Cataudella E., Giraffa C.M., Di Marca S., Pulvirenti A., Alaimo S., Pisano M., Terranova V., Corriere T., Ronsisvalle M.L., Di Quattro R. (2017). Neutrophil-to-lymphocyte ratio: An emerging marker predicting prognosis in elderly adults with community-acquired pneumonia. J. Am. Geriatr. Soc..

[32] Liu X., Shen Y., Wang H., Ge Q., Fei A., Pan S. (2016). Prognostic significance of neutrophil-to-lymphocyte ratio in patients with sepsis: A prospective observational study. Mediat. Inflamm..

[33] Liu S., Wang X., She F., Zhang W., Liu H., Zhao X. (2021). Effects of neutrophil-to-lymphocyte ratio combined with interleukin-6 in predicting 28-day mortality in patients with sepsis. Front. Immunol..

[34] Akilli N.B., Yortanlı M., Mutlu H., Günaydın Y.K., Koylu R., Akca H.S., Akinci E., Dundar Z.D., Cander B. (2014). Prognostic importance of neutrophil-lymphocyte ratio in critically ill patients: Short-and long-term outcomes. Am. J. Emerg. Med..

[35] Ljungström L., Pernestig A.-K., Jacobsson G., Andersson R., Usener B., Tilevik D. (2017). Diagnostic accuracy of procalcitonin, neutrophil-lymphocyte count ratio, C-reactive protein, and lactate in patients with suspected bacterial sepsis. PLoS ONE.

[36] Lowsby R., Gomes C., Jarman I., Lisboa P., Nee P., Vardhan M., Eckersley T., Saleh R., Mills H. (2014). Neutrophil to lymphocyte count ratio as an early indicator of blood stream infection in the emergency department. Emerg. Med. J..

[37] Ng W.W.-S., Lam S.-M., Yan W.-W., Shum H.-P. (2022). NLR, MLR, PLR and RDW to predict outcome and differentiate between viral and bacterial pneumonia in the intensive care unit. Sci. Rep..

[38] Elzey B.D., Sprague D.L., Ratliff T.L. (2005). The emerging role of platelets in adaptive immunity. Cell. Immunol..

[39] Brogly N., Devos P., Boussekey N., Georges H., Chiche A., Leroy O. (2007). Impact of thrombocytopenia on outcome of patients admitted to ICU for severe community-acquired pneumonia. J. Infect..

[40] Wang L., Liang D., Xu X., Jin J., Li S., Tian G., Gao Z., Liu C., He Y. (2017). The prognostic value of neutrophil to lymphocyte and platelet to lymphocyte ratios for patients with lung cancer. Oncol. Lett..

[41] Ouanes Y., Chaker K., Nouira Y. (2023). Prognostic significance of the preoperative platelet-lymphocyte ratio in nonmetastatic renal cell carcinoma: Cross-sectional study. Ann. Med. Surg..

[42] Azab B., Shah N., Akerman M., McGinn J.T. (2012). Value of platelet/lymphocyte ratio as a predictor of all-cause mortality after non-ST-elevation myocardial infarction. J. Thromb. Thrombolysis.

[43] Akboga M.K., Canpolat U., Yayla C., Ozcan F., Ozeke O., Topaloglu S., Aras D. (2016). Association of platelet to lymphocyte ratio with inflammation and severity of coronary atherosclerosis in patients with stable coronary artery disease. Angiology.

[44] Kutlucan L., Kutlucan A., Basaran B., Dagli M., Basturk A., Kozanhan B., Gur M., Senocak E., Kos M. (2016). The predictive effect of initial complete blood count of intensive care unit patients on mortality, length of hospitalization, and nosocomial infections. Eur. Rev. Med. Pharmacol. Sci..

[45] Shen Y., Huang X., Zhang W. (2019). Platelet-to-lymphocyte ratio as a prognostic predictor of mortality for sepsis: Interaction effect with disease severity—A retrospective study. BMJ Open.

[46] Zheng C.-F., Liu W.-Y., Zeng F.-F., Zheng M.-H., Shi H.-Y., Zhou Y., Pan J.-Y. (2017). Prognostic value of platelet-to-lymphocyte ratios among critically ill patients with acute kidney injury. Crit. Care.

[47] Jensen G.L., Rogers J. (1998). Obesity in older persons. J. Am. Diet. Assoc..

[48] Himes C.L. (2000). Obesity, disease, and functional limitation in later life. Demography.

[49] Kaplan M.S., Huguet N., Newsom J.T., McFarland B.H., Lindsay J. (2003). Prevalence and correlates of overweight and obesity among older adults: Findings from the Canadian National Population Health Survey. J. Gerontol. Ser. A Biol. Sci. Med. Sci..

[50] Hanahan D., Weinberg R.A. (2011). Hallmarks of cancer: The next generation. Cell.

[51] Johnson A.R., Justin Milner J., Makowski L. (2012). The inflammation highway: Metabolism accelerates inflammatory traffic in obesity. Immunol. Rev..

